# The “Spiked Helmet Sign” on Electrocardiogram Following Left Ventricular Assist Device Implantation: A Case Report

**DOI:** 10.1111/anec.70237

**Published:** 2026-09-25

**Authors:** Qin Qian, Ling Yin

**Affiliations:** ^1^ Anhui Chest Hospital Hefei China; ^2^ College of Medicine University of Florida Gainesville Florida USA; ^3^ College of Modern Agriculture Weifang Institute of Technology Weifang China

**Keywords:** cardiac tamponade, electrocardiogram, left ventricular assist device, LVAD complications, pericardial hematoma, post‐cardiac injury syndrome, Spiked Helmet Sign

## Abstract

**Background:**

The ECG “Spiked Helmet Sign,” with diffuse concave ST‐segment elevation and a J‐point spike, typically suggests acute pericarditis. However, after LVAD implantation, it may herald a localized pericardial hematoma and impending tamponade.

**Case Presentation:**

A 68‐year‐old male with end‐stage ischemic cardiomyopathy underwent LVAD placement. Hours postoperatively, ECG showed this sign with QTc prolongation (578 ms). Bedside echocardiography revealed a 6‐mm localized pericardial/mediastinal hematoma. Troponin I peaked on day 1 and fell. Conservative management was successful.

**Conclusion:**

Recognition of this ECG sign after LVAD surgery should prompt immediate echocardiography to detect pericardial complications.

## Graphical abstract

The Spiked Helmet Sign on electrocardiogram after LVAD implantation serves as a critical early warning of localized pericardial hematoma and impending tamponade. Prompt recognition should trigger immediate echocardiographic evaluation, as demonstrated in this case, to guide urgent management.

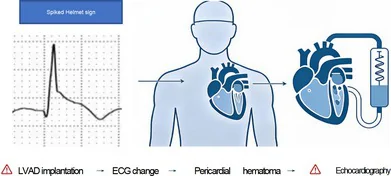

## 1 Introduction

Electrocardiographic (ECG) changes can provide crucial early clues to these complications. The “Spiked Helmet Sign” (also descriptively termed saddle‐back ST elevation) is a distinctive ECG pattern involving diffuse, upwardly concave ST‐segment elevation across multiple leads. While traditionally linked to acute pericarditis (Spodick 2003), this pattern in the postoperative setting is highly suggestive of pericardial irritation from blood or inflammatory exudate, often preceding overt hemodynamic compromise (Alerhand et al. 2022). We present a detailed case of this sign emerging after LVAD implantation, its correlation with imaging findings, and discuss its pathophysiological and clinical implications.

## 2 Case Presentation

### 2.1 Operative Course and Postoperative Timeline

The patient underwent successful implantation of a continuous‐flow left ventricular assist device (HeartMate 3, Abbott) on December 8, 2025 (Mehra et al. 2019). Intraoperative anticoagulation management included intravenous unfractionated heparin administered by weight‐based protocol (target activated clotting time [ACT] > 400 s). Postoperative anticoagulation was transitioned to a continuous heparin infusion, titrated to maintain an activated partial thromboplastin time (APTT) of 40–50 s (Hollis et al. 2024).

### 2.2 Patient Baseline Characteristics

The patient was a 68‐year‐old male with end‐stage ischemic cardiomyopathy. His medical history was notable for prior coronary artery stenting. Preoperative left ventricular ejection fraction was severely reduced at 28%. Coronary angiography performed during preoperative evaluation demonstrated significant in‐stent restenosis with preserved coronary flow (TIMI 3) and no evidence of acute vessel occlusion. He was listed for HeartMate 3 LVAD implantation as destination therapy.

### 2.3 Immediate Postoperative Course (1 h Post‐Procedure)

**FIGURE 1:**
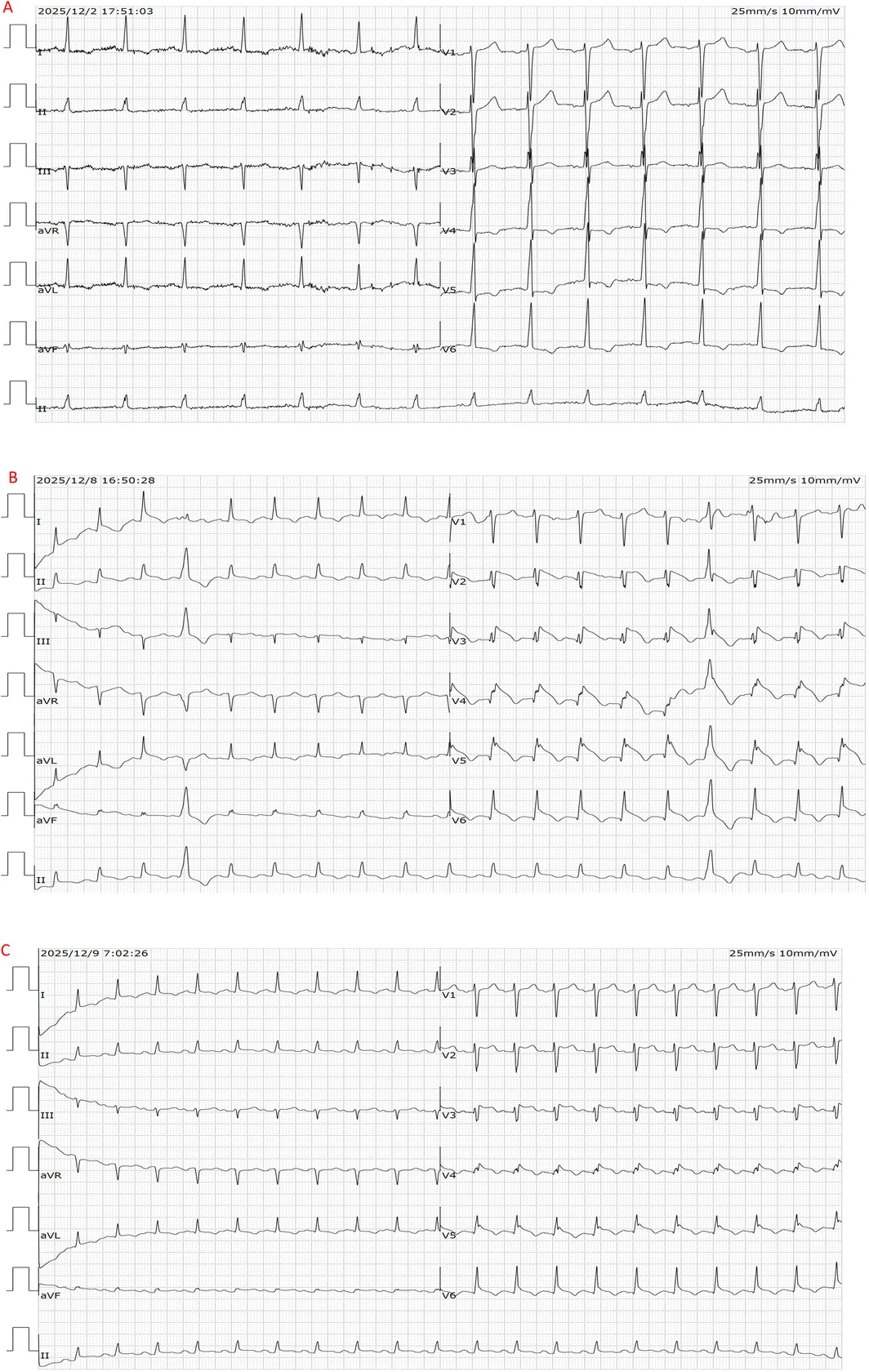
Serial electrocardiograms demonstrating the evolution of the Spiked Helmet Sign. (A) Preoperative ECG (December 2) shows sinus rhythm with nonspecific ST‐T abnormalities (QTc 476 ms). (B) ECG at 1 h post‐LVAD implantation (December 8) reveals sinus tachycardia and diffuse, concave ST‐segment elevation (arrows) in leads V2–V6, II, III, and aVF, characteristic of the Spiked Helmet Sign. QTc is prolonged to 578 ms. (C) Follow‐up ECG on postoperative day 1 (December 9) shows marked resolution of the ST‐segment elevations and normalization of the QTc interval to 478 ms.

The patient's preoperative electrocardiogram demonstrated sinus rhythm with nonspecific ST‐T abnormalities and QTc of 476 ms (Figure 1A). One hour post‐LVAD implantation, ECG revealed sinus tachycardia (115 bpm) with striking, concave ST‐segment elevation in leads II, III, aVF, and V2‐V6, fulfilling the criteria for the “Spiked Helmet Sign” (Figure 1B). Marked QTc prolongation to 578 ms was noted. Initial troponin‐I was elevated at 6.63 ng/mL. Given the concerning ECG findings, urgent bedside transthoracic echocardiography (TTE) was performed.

### 2.4 Diagnostic Findings (Postoperative Day 1)

**FIGURE 2:**
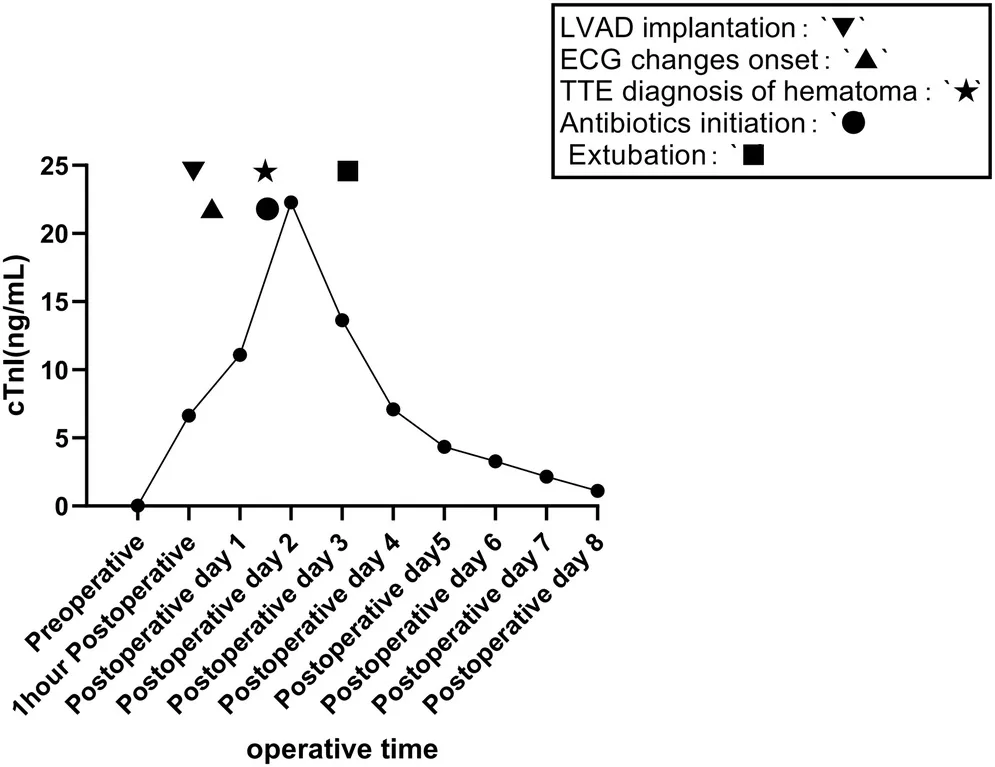
Temporal trend of serum troponin I. The trajectory shows an elevation immediately after surgery (6.63 ng/mL at 1 h), a peak on postoperative day 1 (22.29 ng/mL), and a progressive decline through postoperative day 4 (7.10 ng/mL). Key clinical events are annotated. POD, postoperative day.

Bedside TTE, obtained via subcostal and apical views on the day following surgery, identified a new heterogeneous, moderately echo‐dense collection measuring approximately 6 mm in maximum thickness, localized along the right ventricular free wall within the anterior pericardial/mediastinal space. The collection demonstrated irregular margins and internal echotexture consistent with acute clot formation rather than a simple serous effusion, which would typically appear as an echo‐free space. No right ventricular diastolic collapse, right atrial inversion, or significant respiratory variation across the tricuspid or mitral valves was observed, arguing against hemodynamically significant tamponade physiology at that time. Serial TTEs over the following days confirmed stability of the collection without interval expansion. Real‐time imaging findings were documented in detail in the medical record and communicated directly to the primary team. Serum troponin I peaked at 22.29 ng/mL on postoperative day 1 and subsequently declined to 7.10 ng/mL by postoperative day 4 (Figure 2).

### 2.5 Hospital Course (Postoperative Days 1–4)

Chest radiography revealed endotracheal tube placement, postoperative changes, and bilateral pulmonary infiltrates suggestive of pneumonia. Broad‐spectrum antibiotics (cefoperazone‐sulbactam 1.5 g Q8h + vancomycin 1.0 g Q12h) were initiated pending microbiological results. Serial ECGs documented gradual resolution of the ST‐segment elevations (Figure 1C). QTc normalized to ≈478 ms. Meanwhile, troponin‐I levels decreased to 7.10 ng/mL by postoperative day 4. The patient developed ventilator‐associated pneumonia ( *Klebsiella pneumoniae* ), treated successfully with targeted antibiotics. Serial TTEs showed a stable hematoma without hemodynamic compromise. He was extubated on postoperative day 3 and discharged home on postoperative day 17 after a period of rehabilitation.

## 3 Discussion

This case provides a classic example of the “Spiked Helmet Sign” as a direct electrocardiographic consequence of pericardial hematoma following LVAD surgery.

### 3.1 Pathophysiology: Linking the Sign to the Hematoma

The pathogenesis of this ECG pattern following LVAD surgery is multifactorial. First, the procedure itself—involving apical coring and extensive pericardial manipulation—causes direct epicardial injury, inciting a localized inflammatory response consistent with the post‐cardiac injury syndrome (Kytö et al. 2014). Concurrently, postoperative bleeding into the confined pericardial space forms a hematoma. This collection physically irritates the epicardium, generating diffuse injury currents. Moreover, blood within the pericardium alters the electrical conductivity of the pericardial milieu. The summation of these effects—epicardial irritation, potential compressive ischemia, and altered current conduction—manifests as the characteristic widespread, concave ST‐segment elevation (Spodick 2003; Alerhand et al. 2022). The presence of blood as a conductor, combined with diffuse involvement of the parietal and visceral pericardium, explains why the ST‐segment change is generalized rather than localized. The transient nature of the changes in our patient aligns with a stable, non‐expanding hematoma.

### 3.2 Anatomical Basis for Precordial Lead Predominance

Classically, the “Spiked Helmet Sign” has been described predominantly in the inferior leads (II, III, aVF), typically in the setting of non‐cardiac critical illness associated with elevated intra‐abdominal or intrathoracic pressure (e.g., bowel obstruction, pancreatitis), where diaphragmatic irritation of the inferior pericardium generates a spatially directed injury current. In the present case, while inferior leads were involved, ST‐segment elevation was most pronounced across the precordial leads (V2–V6). This distribution can be mechanistically linked to the anatomical location of the hematoma. The collection was localized along the right ventricular free wall in the anterior pericardial and mediastinal space—directly subjacent to the anterior and lateral chest wall—where it exerted focal mechanical irritation and compressive effects on the underlying epicardium. The resulting injury vector projects anteriorly and laterally, thereby maximizing ST elevation in the overlying precordial leads. In contrast to the systemic, pressure‐mediated inferior predominance seen in abdominal crises, the post‐cardiac‐surgery pattern is spatially determined by the site of localized bleeding and pericardial manipulation. Thus, the lead distribution of the Spiked Helmet Sign may serve as a crude electrocardiographic localizer of the offending pericardial collection.

### 3.3 Clinical Significance: An Early Warning Signal

In the sedated, critically ill postoperative patient, classic signs of tamponade (e.g., hypotension, pulsus paradoxus) are often absent or late findings. The ECG therefore becomes a vital monitoring tool. The “Spiked Helmet Sign” serves as a sentinel marker, potentially appearing before hemodynamic collapse (Alerhand et al. 2022). Its recognition should trigger an immediate bedside echocardiogram to evaluate for pericardial effusion/hematoma and assess for signs of tamponade physiology.

### 3.4 Differential Diagnosis and Management Implications

The primary differential diagnosis for diffuse ST‐segment elevation is acute myocardial infarction (AMI). Key distinguishing features are summarized in Table 1.

**TABLE 1:** Differential diagnosis: Spiked Helmet Sign versus acute myocardial infarction.

Feature	Spiked Helmet Sign (pericardial hematoma/pericarditis)	Acute myocardial infarction
ST‐segment morphology	Concave (“saddle‐shaped”)	Convex (domed)
Distribution	Diffuse, often involving limb and precordial leads	Territorial, corresponding to an occluded coronary artery
Evolution	May resolve gradually without Q‐wave formation	Typically evolves with T‐wave inversion and possible Q‐wave development
Echocardiogram	May show pericardial effusion/hematoma; regional wall motion abnormalities are less specific	Often shows regional wall motion abnormalities corresponding to the infarct zone
Coronary angiography	Usually patent (unless coincident disease)	Identifies an occluded culprit artery

In the present case, several factors collectively argued against acute myocardial infarction and supported a decision not to perform emergent coronary angiography in the immediate postoperative period. First, the ST‐segment elevation was diffuse and upwardly concave across multiple non‐coronary territories, lacking the focal territorial distribution and reciprocal ST depression characteristic of acute plaque rupture. Second, coronary angiography performed during preoperative evaluation had demonstrated preserved coronary flow (TIMI 3) with only in‐stent restenosis and no acute occlusive lesion. Third, the patient had just undergone cardiopulmonary bypass and apical coring; repeat heparinization for invasive angiography in the acute postoperative window would have entailed substantial bleeding risk and potential expansion of the pericardial hematoma. Fourth, the troponin trajectory—peaking within 24 h and declining rapidly thereafter—was consistent with expected perioperative myocardial injury rather than the sustained or late‐peaking pattern of acute infarction. Management therefore hinged on echocardiographic findings. A small, stable hematoma in a hemodynamically stable patient can be managed conservatively with serial imaging, as in this case. Any evidence of tamponade or hemodynamic instability mandates emergent pericardiocentesis or surgical exploration.

### 3.5 Limitations and Generalizability

This report is a single‐case description. The patient's specific characteristics (e.g., advanced age, ischemic cardiomyopathy, prior stenting) may influence both the risk of pericardial hematoma and the clinical course. Although the Spiked Helmet Sign is recognized as a valuable warning sign, its sensitivity and specificity in the LVAD population require validation in larger prospective studies. Nevertheless, the pathophysiological principles illustrated here are broadly applicable to post‐cardiac surgery settings. Additionally, while the sign is highly suggestive, its absence does not rule out a pericardial complication. Furthermore, because repeat invasive coronary angiography was not performed in the immediate postoperative period, periprocedural myocardial ischemia or microinfarction cannot be definitively excluded as a contributing cause of the ST‐segment elevation. Although the diffuse concave morphology, recent patent coronary angiography, typical perioperative troponin trajectory, and echocardiographic confirmation of a localized hematoma collectively favor pericardial irritation as the primary mechanism, the absence of invasive coronary confirmation remains an important limitation of this report. In addition, because the bedside echocardiographic images were not archived, no still‐frame imaging reproduction could be provided in this report. The sonographic findings described here were based on real‐time interpretation by an experienced echocardiographer and documented contemporaneously in the medical record. We acknowledge that the lack of archived images limits the reproducibility of the visual evidence. Future reports of similar cases should ensure standardized acquisition and storage of echocardiographic images at the time of the study.

## 4 Conclusion

The Spiked Helmet Sign on ECG following LVAD implantation is a clinically significant finding that should alert clinicians to the high probability of pericardial hematoma. It is an early, non‐invasive warning sign for potential cardiac tamponade. Proficiency in recognizing this pattern, followed by prompt echocardiographic confirmation and appropriate intervention, is crucial for optimizing outcomes in this high‐risk patient population.

## Author Contributions

Qin Qian: conceptualization, data curation, case collection, writing – original draft, final approval of manuscript, corresponding author; Ling Yin: formal analysis, literature review, writing – review and editing and final approval. All authors have read and agreed to the published version of the manuscript and satisfy all four ICMJE authorship criteria.

## Funding

The authors have nothing to report.

## Consent

Written informed consent was obtained from the patient for publication of this case report and any accompanying images.

## Conflicts of Interest

The authors declare no conflicts of interest.

## Data Availability

The data that support the findings of this study are available on request from the corresponding author. The data are not publicly available due to privacy or ethical restrictions.
